# Emerging minimally invasive laser and light-based therapies for glioblastoma: a systematic review

**DOI:** 10.3389/fonc.2025.1702399

**Published:** 2026-01-20

**Authors:** José Geraldo Medeiros Netto, Lizen Clare André Moreira, Rafaella Mafezoni, Clara Peixoto Cirillo Costa, Lucas Longo Ferreira, Billy McBenedict, Bruno Lima Pessôa

**Affiliations:** Department of Neurosurgery, Antônio Pedro University Hospital, Federal Fluminense University, Niterói, Brazil

**Keywords:** GBM, glioblastoma, grade IV astrocytoma, laser interstitial thermal therapy, LITT, PDT, photodynamic therapy, systematic review

## Abstract

**Introduction:**

Glioblastoma is the most common malignant brain tumor. Standard treatment involves surgical resection with radiotherapy and chemotherapy, but tumors in deep or eloquent brain regions often limit surgical options. Laser interstitial thermal therapy (LITT) and photodynamic therapy (PDT) have emerged as minimally invasive alternatives.

**Materials and methods:**

This systematic review followed PRISMA 2020 guidelines. Study quality was assessed using the Newcastle-Ottawa Scale, ROBINS-I, and RoB 2 tools. Data extraction included tumor characteristics, survival outcomes, quality of life, treatment response, and complications.

**Results:**

A total of 1,468 records were identified; after screening and eligibility assessment, 11 studies involving patients aged 16–86 were included. LITT was found to be safe and effective for recurrent glioblastoma, with 12-month survival rates up to 65%, particularly in patients with small, deep-seated tumors and high Karnofsky scores. PDT, when combined with gross total resection, reduced postoperative edema and improved survival but was associated with higher rates of distant recurrence.

**Discussion:**

LITT and PDT are promising minimally invasive strategies for glioblastoma management, each with distinct mechanisms and clinical roles. LITT is most beneficial for deep-seated, unresectable tumors and may enhance tumor immunogenicity. PDT, though limited by shallow light penetration, effectively eliminates residual tumor cells post-resection and may reduce local recurrence. However, variability in patient selection, tumor features, and treatment protocols across studies limits direct comparison. Adverse events, while generally manageable, require close monitoring. Current evidence supports the adjunctive use of both therapies, but large-scale randomized trials are needed to confirm efficacy, standardize protocols, and explore combinations with immunotherapy.

**Conclusion:**

LITT and PDT are safe and effective adjunct therapies for glioblastoma patients, improving survival and reducing complications in selected patients. LITT mostly benefits patients with small, deep, or unresectable tumors, while PDT enhances outcomes when combined with gross total resection. Further large-scale trials are needed to optimize their use and refine patient selection.

## 1 Introduction

Glioblastoma is the most commonly occurring malignant tumor of the brain and central nervous system (1), characterized by rapid proliferation, diffuse infiltration, and high recurrence rates (2). Its incidence is approximately 3.2 per 100,000 people (1). Glioblastoma belongs to the family of gliomas, which are classified according to the presumed cell of origin (3), and is believed to arise from neural stem cells or lineage-restricted progenitor cells (4).

From a clinical perspective, the current standard of care for newly diagnosed glioblastoma includes maximal safe surgical resection followed by adjuvant radiotherapy (RT) and chemotherapy (CT) with temozolomide (TMZ) (5). However, in many cases, especially when tumors are located in eloquent or deep-seated brain regions, complete resection is not feasible. Consequently, patients ineligible for surgery often experience significantly poorer outcomes (6).

In response to these challenges, minimally invasive laser- and light-based therapies such as laser interstitial thermal therapy (LITT) and photodynamic therapy (PDT) have gained traction in neurosurgical applications for various pathologies, including glioblastoma, low-grade gliomas, and cavernous malformations (7, 8). These techniques aim to maximize local tumor control while minimizing damage to healthy brain tissue.

LITT is a cytoreductive neurosurgical procedure in which focused thermal energy is delivered via a fiberoptic laser probe, inserted through a small burr hole and guided by magnetic resonance imaging (MRI) (9, 10). The laser induces coagulative necrosis of the tumor, with real-time MRI thermometry used to monitor and control ablation in surrounding tissues (11). Compared to open craniotomy, LITT requires only a small incision, which translates to lower risk of wound complications, cerebrospinal fluid fistulae, postoperative pain, as well as shorter recovery times (12, 13). Of particular relevance, LITT can be safely repeated without concerns about cumulative radiation exposure, making it a promising adjunct to standard chemoradiation for managing tumor recurrence or multifocal disease in glioblastoma patients who are ineligible for surgery (13).

Beyond its cytoreductive effect, LITT also exerts immunomodulatory effects, including CD8^+^ T-cell infiltration and increased PD-L1 expression, suggesting it may help reshape the immunosuppressive tumor microenvironment in glioblastoma (14). It has also been shown to transiently disrupt the blood–brain barrier (BBB), potentially enhancing the delivery and efficacy of chemotherapeutic agents (15).

PDT represents another minimally invasive treatment modality that, like LITT, can serve as an alternative or adjunct to standard glioblastoma therapies. PDT uses photosensitizing agents that target cancer cells and are activated by laser light at specific wavelengths, causing the drug to produce toxic molecules that destroy the cancer cells (8). Once activated, the photosensitizer produces reactive oxygen species, primarily singlet oxygen, which induces tumor cell death through oxidative stress (16). As a result, this process can damage cellular membranes, organelles, and DNA, leading to apoptosis, necrosis, or autophagy (17, 18).

Furthermore, in addition to inducing oxidative damage, PDT also disrupts tumor vasculature and can stimulate anti-tumor immune responses (17, 19). In glioblastoma, 5-aminolevulinic acid (5-ALA)-mediated PDT is gaining attention, particularly for its ability to selectively target tumor cells while sparing surrounding healthy brain tissue (20, 21). Once administered, 5-ALA is preferentially taken up by tumor cells and metabolized into protoporphyrin IX (PpIX), a fluorescent and photosensitive compound (22). Upon light activation, PpIX induces localized phototoxic effects, allowing precise control over tumor ablation (17, 21).

Given the limited consensus in the literature regarding the efficacy and safety of laser- and light-based therapies for glioblastoma, this article aims, through a systematic review, to evaluate the potential role of LITT and PDT as alternative or adjuvant treatments to the current standard of care for this fatal disease.

## 2 Materials and methods

This systematic review was conducted in strict accordance with the Preferred Reporting Items for Systematic Reviews and Meta-Analyses (PRISMA) 2020 guidelines (23). PRISMA guided every stage, from the initial formulation of the research question and the development of the systematic search strategy, to the critical appraisal of included studies and the subsequent presentation of findings.

### 2.1 Search strategy and study selection

A comprehensive and systematic literature search was conducted across four major biomedical databases: PubMed, Embase, Scopus, and Web of Science. The search was performed on July 30, 2025, and was limited to studies published within the last five years and written in English, Portuguese, or Spanish. Medical Subject Headings (MeSH), such as ‘glioblastoma’, were used in combination with additional relevant terms, including ‘laser interstitial thermal therapy’ and ‘photodynamic therapy’. All terms were combined using Boolean operators, with PICOS strategy and detailed search strategies for each database provided in **Supplementary Tables 1, 2**.

Studies were eligible for inclusion if they involved patients diagnosed with glioblastoma and investigated LITT or PDT as therapeutic interventions in clinical trials or cohort studies. Exclusion criteria included review articles, editorials, letters to the editor, retracted publications, conference proceedings, and preclinical studies, both *in vitro* and *in vivo*. Case reports, case series, cross-sectional studies, and clinical trials in phase I or II were also excluded to ensure the inclusion of higher-quality clinical evidence.

Title and abstract screening were independently conducted by two reviewers using the Rayyan Systematic Review platform in its most updated version. Full-text eligibility assessment was subsequently performed independently by another pair of reviewers. Any discrepancies at any stage were resolved through discussion until consensus was reached.

### 2.2 Data extraction

Data extraction utilized a standardized form to ensure consistency and accuracy. The extracted information encompassed study characteristics (publication year, study design, sample size), patient demographics (age, sex), and clinical parameters relevant to treatment outcomes. Tumor characteristics (location, histological diagnosis, molecular markers, tumor volume before and after treatment) were recorded when available. Clinical outcomes included overall survival (OS), progression-free survival (PFS), quality of life (QoL), tumor response, and adverse events or complications. Treatment-specific details such as device type, irradiation protocol, number and duration of sessions, frequency of application, administration method, and follow-up duration were also documented.

### 2.3 Quality assessment

The methodological quality of the included studies was assessed using validated tools appropriate to each study design. These included the Newcastle-Ottawa Scale for cohort studies (24), the Risk Of Bias In Non-randomized Studies of Interventions (ROBINS-I) tool for non-randomized intervention studies (25), and the Risk of Bias 2 (RoB 2) tool for randomized controlled trials (26). Each study was appraised independently by two reviewers, ensuring consistency and minimizing subjectivity in the assessment process. Rather than applying cut-off scores, appraisal results were presented in tabular form to transparently highlight methodological strengths and limitations.

## 3 Results

### 3.1 Selection process

**Figure 1:**
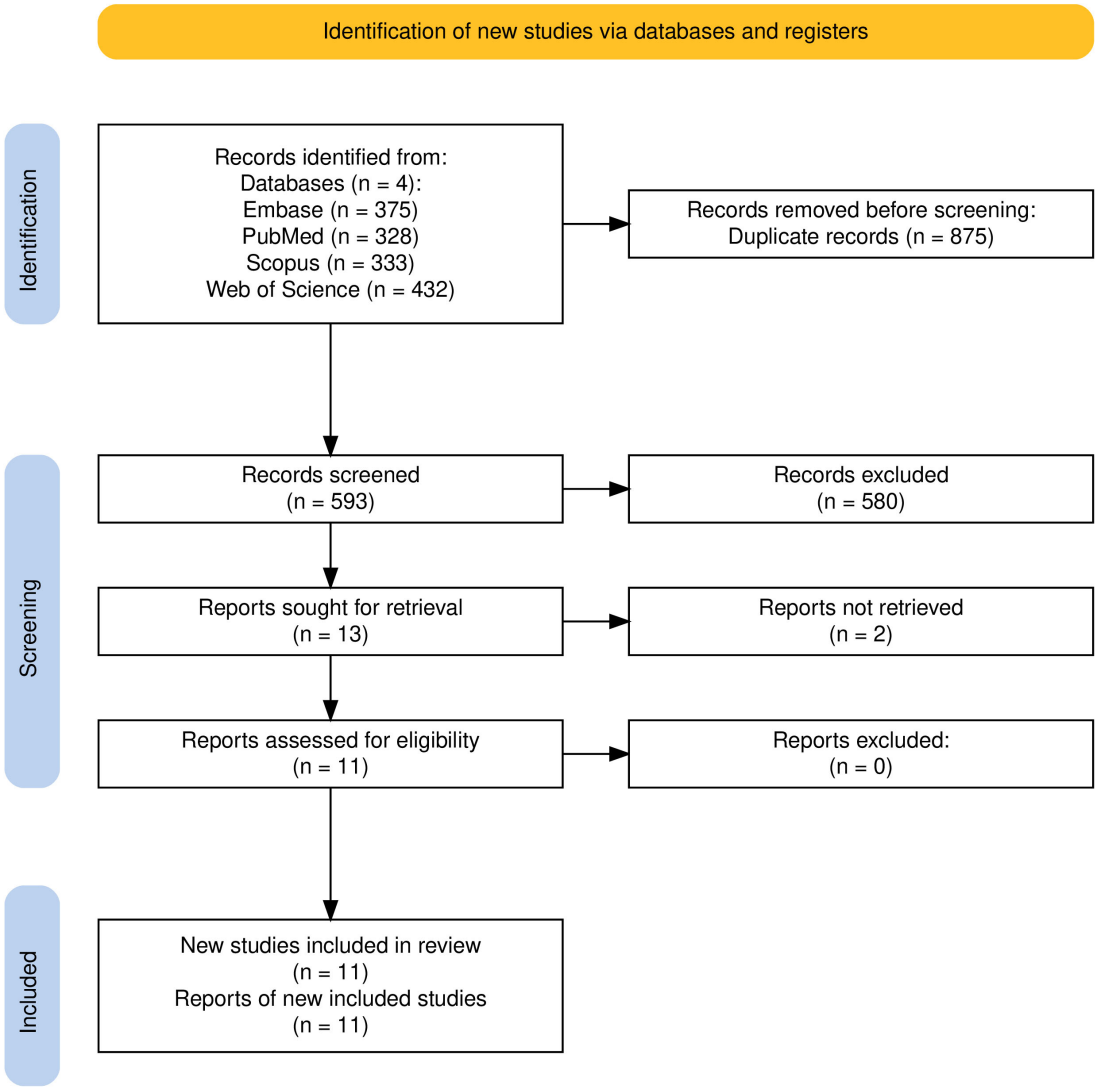
Flow diagram of the number of records identified, screened, excluded, and included at each stage of the systematic review (45).

A total of 1468 entries were identified through database searches, comprising 375 from Embase, 328 from PubMed, 333 from Scopus, and 432 from Web of Science. After removing 875 duplicates, 593 records remained for screening. Of these, 580 were excluded based on title and abstract screening, leaving 13 articles for full-text retrieval and eligibility assessment. Full texts could not be retrieved for 2 studies, and the remaining 11 met the inclusion criteria and were included in the final systematic review. The study selection process is summarized in the PRISMA 2020 flow diagram (**Figure 1**).

### 3.2 Characteristics of included studies

The included studies are detailed in **Table 1**, which summarizes the study designs, populations, interventions, and comparisons. Most employed retrospective designs with cohorts treated using LITT or PDT, and follow-up durations generally exceeded 12 months, adding robustness to the findings.

**Table 1:** Overview of included studies.

Study	Country	Design	Population (n)	Intervention (n)	Comparison (n)
Kobayashi (2022) (31)	Japan	Retrospective cohort	rGBM (108)	Surgery + Intraop. PDT (GTR 64, STR 4, PR 2)	Surgery (GTR 36, PR 2)
Groot (2022) (35)	USA	Non-RCT	nGBM (29); rGBM (60)	LITT (89)	—
Fadel (2022) (33)	USA	Retrospective cohort	rGBM (40)	Surgery + LITT (GTR 10, STR 7)	Surgery (GTR 16, STR 7)
Kaisman-Elbaz (2023) (37)	USA	Retrospective cohort	nGBM (56)	CRT (partial) + LITT (11); CRT (full) + LITT (40)	No treatment (6)
Quach (2023) (29)	Germany	Retrospective cohort	nGBM (126)	Interstitial PDT + CRT (16)	Surgery + CRT (110; GTR 110)
Viozzi (2023) (27)	The Netherlands	RCT	nGBM (15)	Biopsy + LITT (10)	Biopsy (5)
Daggubati (2023) (34)	USA	Retrospective cohort	bGBM (25)	Biopsy + LITT (bilateral 7, unilateral 4)	Biopsy (14)
Silva Jr (2024) (28)	Brazil	Matched-pair cohort	rGBM (22)	Surgery + Intraop. PDT (GTR 7, STR 2, PR 2)	Surgery (GTR 7, STR 2, PR 2)
Peciu-Florianu (2024) (36)	France	Non-RCT	nGBM (10)	Surgery + Intraop. PDT (GTR 6, STR 4)	—
Li (2024) (30)	China	Retrospective cohort	nGBM (56)	Surgery + PDT (GTR 19, STR 15)	Surgery (GTR 11, STR 11)
Fujimoto (2025) (32)	Japan	Retrospective cohort	nGBM (100)	Surgery + Intraop. PDT (44; ≥50% resected)	Surgery (56; ≥50% resected)

RCT, randomized controlled trial; non-RCT, non-randomized controlled trial; nGBM, newly diagnosed glioblastoma; rGBM, recurrent glioblastoma; bGBM, bilateral glioblastoma; GTR, gross total resection; STR, subtotal resection; PR, partial resection; CRT, chemoradiotherapy; LITT, laser interstitial thermal therapy; PDT, photodynamic therapy.

The demographic characteristics of the patient populations, including age and sex distribution, are presented in **Table 2**. The age range of participants across studies was broad, spanning from 16 to 86 years, reflecting the inclusion of both adolescent and elderly patients.

**Table 2:** Patient demographics of included studies.

Study	Group	N	Age (Mean ± SD/Median)	Female (%)
Kobayashi (2022) (31)	Non-PDT	8	43.4 ± 13.3/42 (16–71)	12 (32%)
	PDT	70	46.7 ± 13.3/43.5 (20–80)	31 (44%)
Groot (2022) (35)	nGBM	29	62.8 ± 13.8	9 (31%)
	rGBM	60	59.0 ± 11.0	32 (53.3%)
Fadel (2022) (33)	Non-LITT	23	59.7 ± 12.9/57 [49–68]	11 (48%)
	LITT	14	60.2 ± 11.5/59 [52–67]	7 (41%)
Kaisman-Elbaz (2023) (37)	LITT	56	—/62.1 (31–84)	21 (38%)
Quach (2023) (29)	Non-PDT	—	—/56.1 (17.2–86.6)	—
	PDT	16	59.7 ± 14.2/65.8 (29.7–76.5)	4 (25%)
Viozzi (2023) (27)	Non-LITT	4	59.5 ± 8.5/56 (46–80)	1 (25%)
	LITT	10	62.8 ± 7.3/61 (50–79)	2 (20%)
Daggubati (2023) (34)	Non-LITT	14	—/66.5	10 (71%)
	LITT	11	—/62	1 (9%)
Silva Jr (2024) (28)	Non-PDT	11	49.4 ± 12.5/54 [38–59]	5 (45.5%)
	PDT	11	53.5 ± 10.2/51 [48–57]	5 (45.5%)
Peciu-Florianu (2024) (36)	nGBM	10	56.9 ± 10.3/57.1 [54.5–64.0]	3 (30%)
Li (2024) (30)	STR	26	51.1 ± 14.1	14 (54%)
	GTR	30	52.4 ± 12.8	15 (50%)
Fujimoto (2025) (32)	Non-PDT	56	63.1 ± 12	22 (39%)
	PDT	44	61.5 ± 14	26 (59%)

Age is presented as mean ± standard deviation (SD) and/or median with range or interquartile range (IQR), as reported. Median values are denoted as (range) or [IQR]. nGBM, newly diagnosed glioblastoma; rGBM, recurrent glioblastoma; GTR, gross total resection; STR, subtotal resection; LITT, laser interstitial thermal therapy; PDT, photodynamic therapy.

Regarding LITT, studies demonstrated that this therapy is safe and effective for patients with recurrent gliomas, showing survival rates up to 65% at 12 months. Positive outcomes were associated with good functional status (KPS > 70–80) and tumor volumes less than 10 cm³. The extent of thermal ablation influences results, and treatment is generally well tolerated, with mostly transient morbidities. LITT also showed comparable outcomes to conventional surgery for recurrent glioblastomas, with the advantage of shorter hospital stays and faster recovery. For PDT, studies indicate that adding PDT to tumor resection can reduce postoperative cerebral edema and intracranial pressure, especially when gross total resection (GTR) is achieved. PDT showed significant benefits in PFS and OS compared to conventional treatment, although it may be associated with higher rates of distant dissemination, warranting close monitoring. Molecular profiles, such as MGMT promoter methylation, also proved important prognostic factors in patients undergoing PDT.

Additionally, adjuvant therapies combining RT and TMZ-based CT correlated with improved outcomes, highlighting the importance of multimodal strategies for local control and prolonged survival.

Ilaria Viozzi et al. conducted a study involving 10 patients who underwent LITT (27). A noteworthy aspect of the inclusion process was the use of a Karnofsky Performance Status (KPS) >70 as an eligibility criterion, which demonstrated poorer survival in patients with KPS <80 undergoing LITT. The median age of the sample was 61 years, with a median tumor volume of 9.6 cm³ and a median KPS of 80. Tumor locations included the frontal, parietal, and occipital lobes, as well as involvement of the thalamus and corpus callosum.

During follow-up, eight patients in the LITT group experienced adverse events, including one case of cerebral edema with hydrocephalus and neurological deterioration (KPS <70), and one death related to progressive intracranial bleeding. After three months, nine patients remained alive. Functional decline and worsening QoL were observed, with reductions in KPS scores, increased QLQ-BN20 scores, and decreased EQ-5D and Visual Analogue Scale (VAS) scores. Additionally, follow-up MRI scans performed by eight patients showed increased tumor volume in both T1 with contrast and T2 sequences, in both the LITT and control groups.

Silva Jr. et al. conducted a study involving 11 patients with recurrent high-grade glioma treated with 5-ALA fluorescence-guided resection combined with intraoperative PDT (28). The median age of participants was 51 years. PDT required an additional surgical time of 70 to 80 minutes (median of 73 minutes), including 60 minutes of irradiation divided into five fractions with four 2-minute intervals, in addition to setup time. A linear power density of 200 mW/cm and an energy density of 720 J/cm were applied in all cases. Among the reported adverse events, two patients developed cerebrospinal fluid fistula followed by meningitis, one patient experienced a surgical site infection, and another presented with symptomatic cerebral edema and obstructive hydrocephalus, requiring a ventriculoperitoneal shunt.

During clinical follow-up, four patients died, and four experienced local progression within six months after treatment. One patient had local recurrence at 10 months, and another showed disease progression at three months. In contrast, five patients remained alive and progression-free at the last follow-up.

Analysis of adjuvant therapies suggests a possible correlation with clinical outcomes. Patients who underwent combined therapeutic strategies — such as RT with TMZ, Gamma Knife followed by bevacizumab, or RT alone — remained alive and without tumor progression. In comparison, among those treated with bevacizumab alone, one experienced late local recurrence (10 months) and remained alive, while another had early disease progression. These findings suggest that more aggressive or multimodal adjuvant regimens may be associated with improved local control and PFS. However, the small sample size limits definitive conclusions and highlights the need for further prospective studies.

Stefanie Quach et al. reported a cohort of 16 patients consecutively treated with interstitial PDT (29). The median age at the time of treatment was 65.8 years, and all patients had a KPS of 90. The tumors were primarily located in the temporal lobe, followed by the parietal, frontal lobes, and the central gyrus/subcentral region. The median tumor volume was 6.1 cm³. Regarding the molecular profile, 50% of patients (8/16) exhibited MGMT promoter methylation, while 12.5% (2/16) harbored IDH mutations. The median PFS was 16.4 months, and the median OS reached 28.0 months, with 13 patients dying during the follow-up period. One- and two-year PFS rates were 56.3% and 43.8%, respectively, while OS rates at the same time points were 75% and 62.5%. Univariate regression analysis showed that MGMT promoter methylation was a significant prognostic factor for both OS (p = 0.04) and PFS (p = 0.04), unlike age, which was not statistically associated with survival outcomes (p = 0.07 for OS; p = 0.67 for PFS).

Within the postoperative period (30 days), 37.5% of patients experienced transient morbidity, including new-onset aphasia, worsening of pre-existing aphasia, new hemiparesis, worsening of pre-existing hemiparesis, and one case of pulmonary embolism. Two patients presented with multiple symptoms. Most neurological complications were associated with cerebral edema and responded well to oral dexamethasone treatment. Only one patient developed permanent aphasia, representing the sole persistent morbidity recorded.

Jingxuan Li et al. analyzed a cohort of 56 patients with glioblastoma, divided into two groups: 30 patients underwent GTR, and 26 underwent subtotal resection (STR), with a mean age of 51.8 years (30). No significant differences were observed between the groups regarding preoperative or intraoperative tumor volumes. The results demonstrated that the volume of cerebral edema was significantly lower in the GTR group compared to the STR group, with peaks of edema and increased intracranial pressure occurring within the first few days and between postoperative days 7 and 10. Patients in the STR group exhibited higher postoperative cerebral edema volumes, suggesting that a greater extent of resection may reduce the adverse biological effects of PDT.

Furthermore, intracranial pressure was significantly lower in the GTRG compared to the STR group during postoperative days 7–10 (p < 0.05). Following combination with PDT, both the absolute values and fluctuations of intracranial pressure were higher in STR group patients compared to those who underwent surgery alone. These findings indicate that GTR combined with PDT is associated with a lower impact on intracranial pressure, whereas STR combined with PDT is more likely to lead to the development of intracranial hypertension.

Additional analysis revealed that larger tumor volumes and greater preoperative peritumoral edema, as well as MGMT promoter methylation, were associated with increased postoperative cerebral edema and elevated intracranial pressure. Lastly, statistical analysis showed that tumor location did not significantly affect clinical symptoms, regardless of whether patients underwent GTR or STR.

Tatsuya Kobayashi et al. conducted a study involving 108 patients, of whom 70 were assigned to the PDT group and 38 to the control group (31). The mean ages were 43.5 and 42 years, respectively, with no statistically significant differences in age, sex, or preoperative KPS. In the PDT group, 69 patients were diagnosed with glioblastoma and one with gliosarcoma, while all patients in the control group had glioblastoma. The IDH1 R132H mutation was identified in 22.9% of the PDT group and in 39.5% of the control group. The median Mib-1 proliferation index was 17.0 (range 1.6–51.4) in the PDT group and 20.7 (range 4.0–46.8) in the control group, with no statistically significant difference (p = 0.11). Regarding safety, adverse events were observed in 4.3% of patients in the PDT group, including one case of wound dehiscence (grade 3), one case of cerebrospinal fluid leakage (grade 2), and one case of acute epidural hematoma (grade 3), all of which required medical intervention. No adverse events were reported in the control group.

The most notable finding of the study was the survival benefit associated with PDT. The median PFS after surgery for recurrence was significantly longer in the PDT group (5.7 months; 95% CI: 3.4–7.1) compared to the control group (2.2 months; 95% CI: 1.5–4.0), with p = 0.0043. Similarly, the median OS in the PDT group was 16.0 months, with 1-year and 2-year survival rates of 73% and 37.4%, respectively, whereas in the control group, the median OS was 12.8 months, with 1-year and 2-year survival rates of 58.8% and 11.5%, respectively — a statistically significant difference (p = 0.031).

Yosuke Fujimoto et al., conducted a study involving the group undergoing PDT included 44 patients, while the non-PDT group comprised 56 patients (32). Final analysis was not possible in 4 cases from the PDT group and 1 case from the non-PDT group. There were no statistically significant differences between the groups in terms of age, tumor volume, MGMT promoter methylation status, or extent of resection.

According to the authors, the median follow-up duration was 23.9 months for the PDT group and 22.5 months for the non-PDT group. Tumor recurrence was observed in 89% of the PDT group and 100% of the non-PDT group. A detailed analysis of recurrence patterns showed that local recurrence was significantly lower in the PDT group compared to the non-PDT group (56.4% *vs*. 83.9%), while distant recurrence and dissemination were significantly higher in the PDT group (48.7% *vs*. 16.1%; p = 0.0033). The authors concluded that PDT provides good local control but may be associated with higher rates of dissemination. However, they emphasized that PDT does not itself promote dissemination, as the time to dissemination did not differ significantly between groups (p = 0.44).

The average number of irradiation points in the PDT group was 13.8 (range: 1 to 35), and this number was significantly associated with tumor volume (p < 0.0001, R² = 0.04). Larger tumor volumes were associated with fewer irradiation points due to limitations such as deep lesions or the presence of blood vessels within the resection cavity, which reduce PDT effectiveness. To assess this, the authors adopted an arbitrary cut-off value of 4 mL per irradiation point (tumor volume divided by number of irradiation points), although no justification for this threshold was provided. They found that patients with less than 4 mL per irradiation point tended to have longer PFS and OS.

Adverse events observed in the PDT group included one case of venous infarction due to irradiation near the portal vein, one case of cerebral edema with hemiparesis, one case of cyst formation in the resection cavity, and one case of delirium.

Regarding prognostic factors, the median PFS was 10.8 months in the PDT group and 9.3 months in the non-PDT group, significantly favoring the PDT group (p = 0.016). Median OS was 24.6 months in the PDT group versus 17.6 months in the non-PDT group, also significantly higher in the PDT group (p = 0.034). Furthermore, PDT treatment and tumor involvement of the subventricular zone were significant prognostic factors. Among patients with subventricular zone involvement and age over 70, survival was significantly reduced. However, in multivariate analysis, age alone was not independently associated with prognosis.

Hassan A. Fadel et al. included 40 patients with unifocal, lobar, first-recurrence glioblastoma in their study: 17 were treated with LITT and 23 underwent surgery, with patients presenting deep-seated lesions being excluded (33). In the LITT group, the median tumor volume was 4.4 cm³, with an ablation extent of 90.7%, and 59% of patients achieved complete ablation. The median preoperative KPS for this group was 90, and all patients were followed for a median of 12.8 months.

In the surgical group, the median tumor volume was 7.54 cm³, with a mean resection extent of 95.1%, and 70% of patients achieved GTR. The median preoperative KPS was also 90, with a median follow-up of 13.8 months. Among patients who underwent surgery, 96% died during follow-up, 5 required reoperation, and no cases of surgical site infection were reported. In the LITT group, 94% died, 1 patient underwent a second LITT procedure, and 5 were submitted to surgery after LITT failure.

Median OS was 14.1 months in the LITT group and 13.8 months in the surgical group. PFS was 3.7 months for LITT and 3.3 months for surgery. Hospital length of stay was significantly shorter for the LITT group (2.2 ± 2.1 days) compared to the surgical group (3.0 ± 1.1 days, p = 0.004).

The authors concluded that LITT for unifocal, lobar recurrent glioblastoma demonstrated survival and safety outcomes comparable to repeat surgery, with the added benefit of shorter hospital stays and faster recovery.

Lekhaj C. Daggubati et al. included 25 patients in their study, of whom 11 underwent a combination of biopsy and LITT, while 14 underwent biopsy alone (34). The majority of participants (21 patients) were diagnosed with glioblastoma, with 10 of these belonging to the biopsy + LITT group.

The median age in the biopsy + LITT group was 62 years, and the most frequently observed symptom was mental confusion. The preoperative KPS was 80 in this group, and 18.18% of the patients had methylation of the MGMT promoter, a positive prognostic marker. Surprisingly, no postoperative complications were reported in the biopsy + LITT group, whereas a complication rate of 3.45% was observed in the biopsy-only group. Notably, all patients maintained their baseline neurological status after the procedure.

In the volumetric analysis, the mean preoperative tumor volume was 32.17 cm³ in the biopsy-only group and 27.15 cm³ in the biopsy + LITT group. The average ablation extent in the LITT group was 73.84%, and the authors reported a significant negative correlation between preoperative tumor volume and ablation extent (p = 0.027), suggesting that smaller tumors had a relatively higher ablation rate.

Regarding clinical outcomes, a significant improvement in survival was observed in the LITT-treated group. PFS was 2.8 months in the biopsy-only group and 5.5 months in the biopsy + LITT group (p = 0.026). OS was 4.3 months in the biopsy-only group and 10.3 months in the biopsy + LITT group (p = 0.035).

Groot et al. reported a study involving 89 patients, of whom 60 had recurrent glioblastoma, 6.7% of whom had received LITT, and 29 were newly diagnosed adult patients, of whom approximately 53.8% initiated RT and TMZ-based CT after LITT (35). The mean age of the 89 patients was 60.2 years. The main reason for using LITT, as identified by the center, was that the lesion was unresectable via traditional surgical approaches in 52% of newly diagnosed patients and 42% of recurrent cases.

The authors reported that most tumors treated with LITT in the newly diagnosed cohort were deeply located, with lesion volumes greater than 3 cm³ in 51.6% of newly diagnosed patients and in 60.8% of recurrent cases. The median laser time was 26 minutes and 32 seconds for newly diagnosed cases and 25 minutes and 8 seconds for recurrent cases, while the median hospital stay was 50 hours. Additionally, the authors described adverse events consistent with those already reported in the literature, including neurological deficits, edema, aphasia, and venous thrombosis. However, one adverse event reported by them stood out — a permanent motor deficit in one patient following ablation of a right frontal lesion adjacent to the precentral gyrus.

Finally, the median OS was 9.73 months (95% CI: 5.16–15.91) and the median PFS was 5.92 months (95% CI: 3.65–not reached). A statistically significant OS benefit was observed in newly diagnosed glioblastoma patients who received both RT and CT within 12 weeks after LITT (16.14 months, 95% CI: 6.11–not reached) compared with those who received only one modality or no treatment after LITT (5.36 months, 95% CI: 2.14–7.69).

Iulia Peciu‐Florianu et al. described a study including ten patients with surgically accessible lesions suitable for maximal resection (36). The median age was 57.1 years [35–69.3], 70% of the patients were male, and the median KPS score was 85 (range 70–100). All patients had histologically confirmed glioblastoma, with IDH mutation present in only one patient, and four patients showed hypermethylation of the MGMT promoter gene.

During the trial, most adverse events (AEs) were attributed to CT and RT (59/167), unspecified causes (14/167), surgery unrelated to the PDT procedure, and/or glioblastoma-related symptoms (13/167). Four AEs were specifically related to the study procedure, linked to 5-ALA HCl intake (1 erythema [grade 2] and 3 liver enzyme elevations [two grade 2, one grade 1]). Seven serious adverse events (SAEs) were reported in five patients, including one death, although the authors did not attribute these events to the PDT procedure. One patient experienced partial status epilepticus associated with a remote relapsing glial lesion, leading to death due to tumor progression.

Regarding efficacy, the median PFS was 16.2 months (95% CI: 10.2–21.4), and the median OS was 28.0 months (95% CI: 21.4–34.6) for all patients treated with PDT in addition to maximal surgical resection and the standard Stupp protocol. These findings suggest that the addition of PDT may significantly prolong both PFS and OS compared to historical data from the Stupp protocol alone.

### 3.3 Risk of bias appraisal

**Figure 2:**
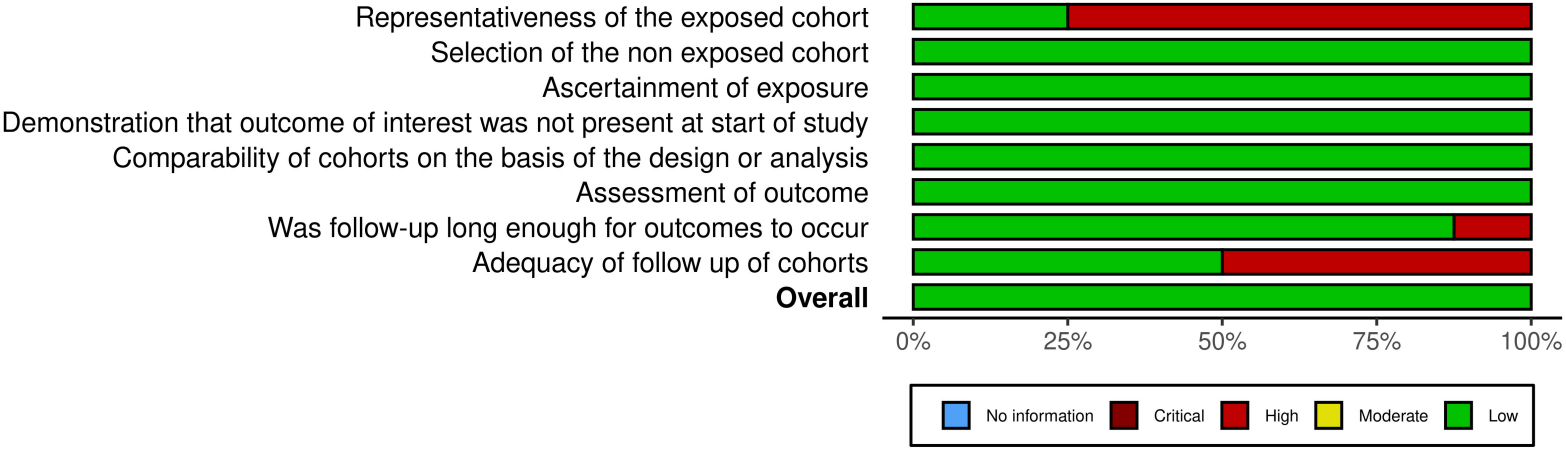
Summary of risk of bias assessment for the included cohort studies using the Newcastle-Ottawa Scale (46).

The risk of bias for the included cohort studies was assessed using the Newcastle-Ottawa Scale. **Figure 2** summarizes the evaluations across all domains: representativeness of the exposed cohort, selection of the non-exposed cohort, ascertainment of exposure, demonstration that the outcome of interest was not present at the start of the study, comparability of cohorts, assessment of the outcome, follow-up duration, and adequacy of follow-up.

In terms of representativeness of the exposed cohort, Li (2024) and Kaisman-Elbaz (2023) were the only studies classified as having a low risk of bias (high representativeness), while all other studies were rated as having higher risk (30, 37). For selection of the non-exposed cohort, all studies were rated low, indicating that the non-exposed cohort was drawn from the same community as the exposed cohort, which minimizes selection bias.

Regarding ascertainment of exposure and demonstration that the outcome of interest was not present at the start of the study, all studies were rated low, as exposure was well documented (e.g., secure records), and outcomes were not present at baseline in all cohorts. Similarly, comparability of cohorts was rated low for most studies, indicating sufficient control for confounding variables in either the design or analysis phases. The exception was Quach (2023), which was rated as moderate risk due to less robust adjustment for potential confounders (29).

In the domain of assessment of outcome, most studies used secure records, while a few employed independent, blind assessments, leading to a low risk of bias in this domain as well. However, concerns arose regarding the follow-up duration and the adequacy of follow-up. While Kobayashi (2022), Quach (2023), Daggubati (2023), and Fujimoto (2025) were classified as having high risk of bias in follow-up adequacy due to insufficient reporting or loss to follow-up, the remaining studies reported complete follow-up and were classified as low risk of bias in this domain (29, 31, 32, 34).

**Figure 3:**
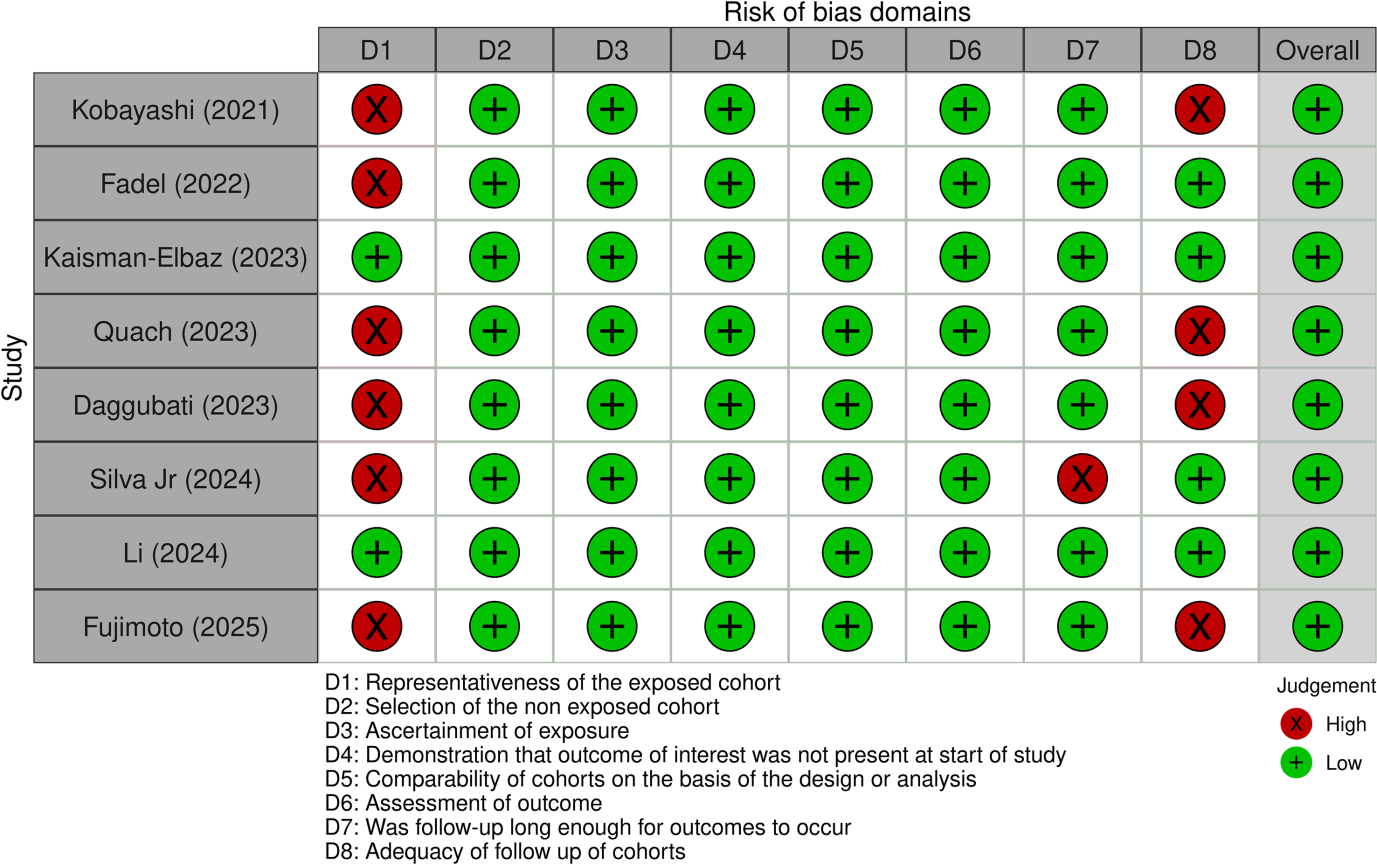
Traffic light plot representing the risk of bias for each cohort study, based on the Newcastle-Ottawa Scale (46).

Overall, despite variability in certain domains, most cohort studies were rated as having a low overall risk of bias. Quach (2023) received a moderate overall rating due to moderate risk in comparability, and high risk in both follow-up adequacy and representativeness of the exposed cohort (29). These findings are illustrated in **Figure 3**, which provides a traffic light plot representing the risk of bias across the cohort studies.

**Figure 4:**
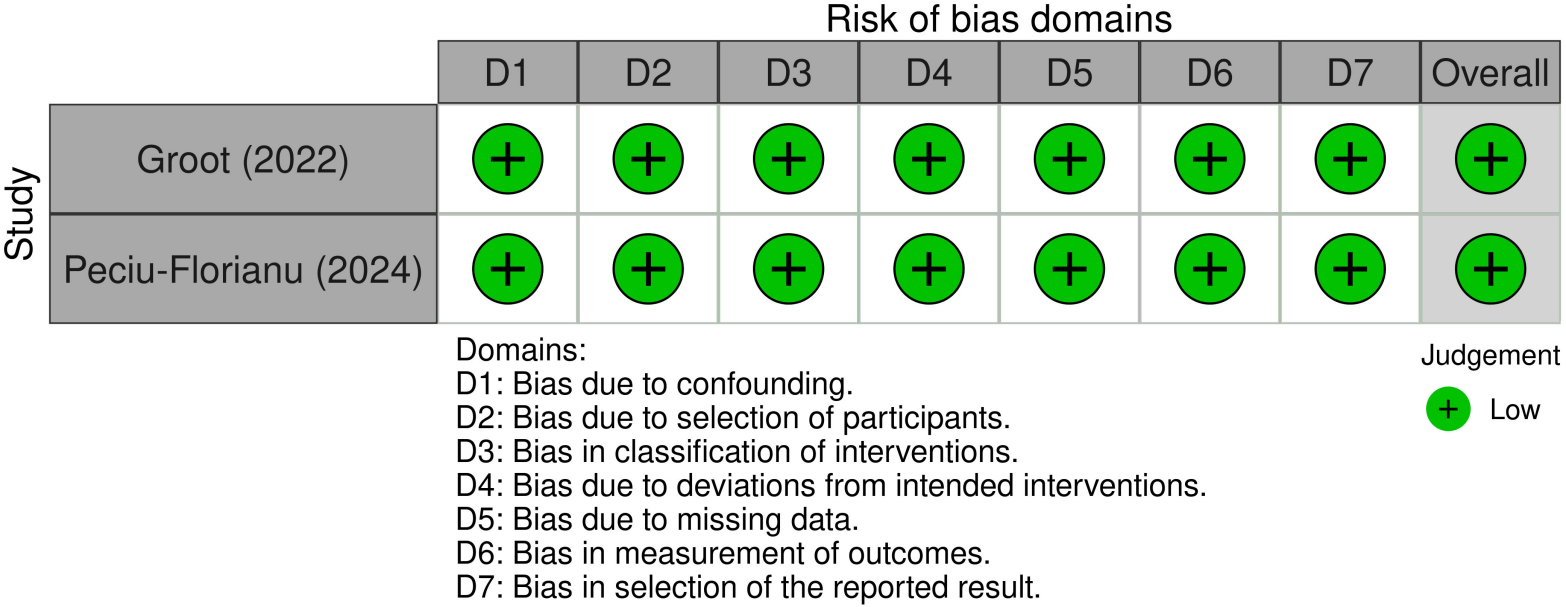
Risk of bias assessments for non-randomized studies, visualized in a traffic light plot using the ROBINS-I tool (46).

For the non-randomized studies, Groot (2022) and Peciu-Florianu (2024) were assessed using the ROBINS-I tool, and both studies were rated as low risk of bias across all domains (35, 36). Both studies demonstrated low risk in all seven domains of the ROBINS-I tool, including biases related to confounding, selection, and measurement of outcomes. As a result, these two studies were classified as having a low overall risk of bias. **Figure 4** illustrates the traffic light plot for the non-randomized studies, showing the ROBINS-I assessments for each.

**Figure 5:**
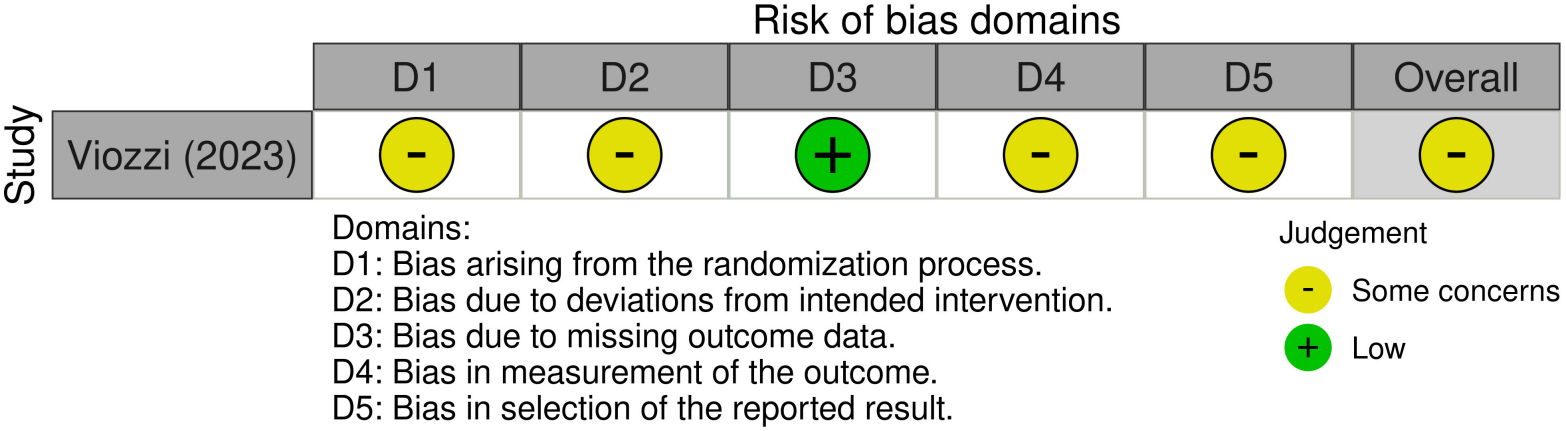
Traffic light plot depicting risk of bias for the randomized controlled trial, assessed through the RoB 2 tool (46).

The only RCT included in this review, Viozzi (2023), was assessed using the RoB 2 tool (27). Some concerns were identified across multiple domains, primarily due to limited reporting on the randomization process, lack of blinding, and potential selective reporting of outcomes. Despite these concerns, the study adhered to the intended intervention, exhibited acceptable levels of missing data, and employed appropriate statistical analyses. Overall, the risk of bias was classified as ‘some concerns’ (**Figure 5**).

## 4 Discussion

The selected studies provide a comprehensive overview of the role of laser-based therapies, including LITT, and PDT, in the treatment of brain tumors, particularly glioblastoma and other high-grade gliomas. These therapies offer minimally invasive alternatives to traditional surgical resection, with potential benefits such as reduced morbidity, shorter hospital stays, and enhanced immune responses. Below, we synthesize the findings from these studies and discuss their implications for clinical practice and future research.

### 4.1 LITT for glioblastoma and metastatic tumors

Recent scientific studies highlight the immunomodulatory potential of LITT. It is well established in the literature that significant infiltration of CD8+ T cells, accompanied by increased PD-L1 expression and reduced corticosteroid use, indicates that LITT can promote sustained tumor immunogenicity (38). This capacity was corroborated by the technique described by Chandar et al. (2023), which demonstrated increased intratumoral mutational burden, neoantigen generation, and persistent induction of immunogenic cell death (14).

Furthermore, findings from the study by Yihe Wang et al. indicated that factors associated with improved survival included KPS > 80 (p = 0.03); tumor volume < 10 cm³, with a median survival of 23 months compared to 5 months in patients with tumors > 10 cm³ (95% CI: 0.06–0.76 *vs*. 1.31–16.14; p = 0.02); and the use of adjuvant therapy following MRgLITT (39). The finding related to KPS suggests, to some extent, a possible selection bias in studies that adopt as inclusion criteria patients with KPS > 70, as proposed by Ilaria Viozzi et al. (27). However, Viozzi observed that after LITT application, there was functional decline and worsened QoL in their sample, reflected by reduced KPS scores. Additionally, Wang et al. identified significant survival differences according to the therapeutic regimen used (p = 0.04), an aspect not evidenced in other analyzed studies (39).

Tehila Kaisman-Elbaz et al. reported that ablation extent was proportional to tumor volume, presenting a median of 84%, with 24 patients achieving ablation extent ≥ 90% (37). Median PFS was 4.8 months (range: 3.5–6.7) and median OS was 10.1 months (range: 7.1–14.8). Larger tumors tended to have lower ablation extent, while higher ablation extent was associated with better survival outcomes, a finding also corroborated by Wang et al. (39).

Another relevant finding related to tumor volume was demonstrated by Yosuke Fujimoto et al., who showed that patients with tumor volume ≥ 10 cm³ had significantly greater peritumoral edema (p < 0.001) and a higher likelihood of developing neurological side effects compared to those with tumors < 10 cm³ (36% *vs*. 13%, p = 0.035) (32).

Complementarily, Daggubati et al. observed that in the biopsy plus LITT group, the mean preoperative tumor volume was 27.15 cm³, and the mean ablation extent was 73.84% (34). A significant negative correlation was found between preoperative tumor volume and ablation extent (p = 0.027), suggesting that smaller tumors had a higher relative ablation rate. This finding is important, as none of the other studies explicitly correlated smaller volumes with higher ablation rates. Finally, Wang et al. highlighted that one of the main factors influencing OS is the presence of a single lesion subjected to ablation, with a median survival of 16.5 months, higher than that observed in patients with multiple lesions (11 months; p = 0.03), reinforcing the impact of tumor volume and lesion number on therapeutic response (39).

Among the main adverse effects observed with the use of LITT are cerebral edema associated with hydrocephalus and neurological deterioration (KPS < 70), as well as one death related to progressive intracranial hemorrhage. After three months, nine patients remained alive. Functional decline and worsening QoL were recorded, evidenced by reductions in KPS scores, increases in QLQ-BN20 scores, and decreases in EQ-5D and VAS scores, as reported by Viozzi (27). The latter described early complications within the first 30 days, including one case of cerebral edema, two cases of altered mental status, one case of hydrocephalus, one intracranial hemorrhage, one seizure, and one urinary tract infection. Late complications included one case of cerebral edema, two of altered mental status, one deep vein thrombosis, one balance disorder, one case of non-communicating hydrocephalus, and one episode of sinus bradycardia. Additionally, Fujimoto identified adverse effects consistent with these reports, highlighting that patients with tumor volumes ≥ 10 cm³ had significantly greater peritumoral edema (p < 0.001) and a higher likelihood of developing neurological effects compared to patients with tumors < 10 cm³ (36% vs. 13%, p = 0.035) (32). Reported adverse effects included altered mental status, neurological deficits, seizures, and refractory headaches.

Finally, it was observed that among the included studies using LITT, none demonstrated that tumor location was a statistically significant factor for increased adverse effects or worsened survival rates. However, it is noted that if tumor location requires a greater extent of ablation, this could impact these parameters.

### 4.2 PDT for brain tumors

PDT, which acts through the use of photosensitizers such as talaporfin sodium in combination with laser irradiation, has demonstrated well-established efficacy in the literature for inducing oxidative stress and promoting selective tumor cell death. However, the limited penetration depth of light into brain tissue, approximately 2 to 4 mm, requires maximal surgical resection of the tumor prior to PDT application, which restricts its use in tumors located in deep regions or eloquent areas of the brain. Despite this limitation, PDT’s ability to eradicate residual tumor cells in the surgical bed represents an important strategy for local disease control, especially in cases of recurrence or in patients refractory to RT.

To illustrate this approach, we methodologically selected the study by Jingxuan Li et al., who evaluated 56 patients divided into two groups undergoing either GTR or STR, both treated with PDT (30). The results showed that cerebral edema volume was significantly lower in the GTR group compared to the STR group, with peaks of edema and increased intracranial pressure occurring in the first few days and between postoperative days 7 and 10. Patients undergoing STR had larger volumes of postoperative cerebral edema, suggesting that a higher extent of resection may mitigate the adverse biological effects of PDT, corroborating findings previously described in the literature.

Another factor that directly impacts PDT efficacy is the tumor’s molecular profile. Consistent with this, Quach et al. reported that 50% of patients (8/16) exhibited MGMT promoter methylation, while 12.5% (2/16) harbored IDH mutations (29). In univariate regression analysis, MGMT promoter methylation was identified as a significant prognostic factor for OS (p = 0.04) and PFS (p = 0.04). These findings align with those of Jingxuan Li et al., who demonstrated that, besides larger tumor volumes and more extensive preoperative peritumoral edema, MGMT promoter methylation was associated with increased postoperative cerebral edema and intracranial pressure (30).

Regarding adverse events associated with PDT, reports from the included studies reveal some common and some specific manifestations. Among the most frequently observed adverse effects are cerebral edema, altered mental status, and neurological complications such as hemiparesis and seizures. Specifically, Kobayashi described cases of operative wound dehiscence (grade 3), cerebrospinal fluid fistula (grade 2), and acute epidural hematoma (31). Fujimoto reported adverse events including venous infarction due to irradiation near the portal vein, cerebral edema with hemiparesis, cyst formation in the resection cavity, and episodes of delirium (32). Quach documented transient morbidity, including newly onset aphasia, worsening of pre-existing aphasia, new or worsened hemiparesis, and one case of pulmonary embolism (29). Thus, the most recurrent adverse effects among the authors are cerebral edema, altered mental status, and transient neurological deficits, reflecting PDT’s impact on the surrounding brain tissue.

One of the most significant findings of this review was presented by Fujimoto et al., who detailed tumor recurrence patterns following PDT (32). Local recurrence was significantly lower in the PDT-treated group (56.4% *vs*. 83.9%), while distant recurrence and tumor dissemination were significantly higher in the same group (48.7% *vs*. 16.1%; p = 0.0033). Nevertheless, the authors concluded that PDT provides good local disease control but may be associated with higher rates of dissemination. However, they emphasized that PDT does not directly promote tumor dissemination, as the time to dissemination did not differ significantly between groups (p = 0.44).

### 4.3 Comparison between LITT and PDT

The applications of LITT and PDT in glioblastoma management differ substantially in terms of patient selection, mechanisms of action, tumor location suitability, and procedural logistics. **Table 3** provides a structured, evidence-based comparison of these two modalities.

**Table 3:** Key clinical distinctions between LITT and PDT in glioblastoma patients.

Therapy	Ideal patient profile	Tumor location	Surgical/resection status	Mechanism & sequelae	Practical considerations
LITT	Patients with small, deep-seated, or recurrent high-grade gliomas not amenable to open resection; also elderly or high-risk patients (7, 40, 41)	Typically deep-seated (thalamus, insula, corpus callosum); difficult or unsafe to reach with open surgery (7, 41)	Not suitable for gross total resection; often used for residual or recurrent disease and where reoperation is high risk (40, 43)	MRI-guided thermal ablation causes targeted coagulative necrosis, often associated with peritumoral edema and transient blood-brain barrier disruption; edema can be substantial, usually manageable with steroids (40, 43)	Requires intraoperative MRI or thermometry, minimally invasive, generally short hospital stay, repeatable in select circumstances (7, 40)
PDT	Patients with resectable, superficial, or cavity-accessible gliomas — often after maximal safe resection, for microscopic or minimal residual disease (42)	Tumor cavity post-resection or superficial tumors; also possible via interstitial light fibers for select deep lesions (42)	Most often post-gross total resection or subtotal resection when a cavity is available for light delivery (42)	Photochemical cell death induced by reactive oxygen species; vascular shutdown and immune effects; edema usually milder and more localized than LITT, but significant if high-dose or large treated volume (42)	Requires pre-op photosensitizer (e.g., 5-ALA), intraoperative illumination planning, strict post-op light avoidance for days, specialist equipment and staff (42)

LITT, laser interstitial thermal therapy; PDT, photodynamic therapy; MRI, magnetic resonance imaging; BBB, blood-brain barrier; GTR, gross total resection; STR, subtotal resection; 5-ALA, 5-aminolevulinic acid; ROS, reactive oxygen species.

#### 4.3.1 Ideal patient profiles

LITT is most appropriate for patients with small, deep-seated, or recurrent high-grade gliomas who are not candidates for open resection, commonly when lesions are located in eloquent or surgically inaccessible brain regions or for elderly/high-risk surgical patients (7, 40, 41). Conversely, PDT is favored in patients with tumors that are surgically accessible or for those with a post-resection cavity amenable to illumination, often when microscopic residual disease is suspected after maximal safe resection (42).

Patients with MGMT promoter methylation tend to exhibit a better prognosis, which may contribute to the observed survival benefits with both LITT and PDT. However, this favorable prognosis is likely attributed to the molecular characteristics of the tumor rather than the therapies themselves. Therefore, while these therapies show potential benefits, the observed outcomes should be interpreted with caution, as the influence of MGMT methylation on therapy response remains an area requiring further investigation.

#### 4.3.2 Mechanisms of action and sequelae

LITT achieves cytoreduction via highly localized thermal ablation, leading to targeted coagulative necrosis. Common post-procedure effects include peritumoral edema and transient blood-brain barrier disruption, both of which can generally be managed with corticosteroids (40, 43). PDT, by contrast, utilizes activation of tumor-selective photosensitizers with light, generating reactive oxygen species and subsequent photochemical tumor cell death, as well as local vascular effects and immune modulation. Edema from PDT is typically milder than that with LITT, although more pronounced effects may occur when treating larger cavities or with high light dosages (42).

#### 4.3.3 Tumor location and treatment efficacy

LITT was preferentially used for deep-seated and eloquent or inaccessible lesions (such as the thalamus, insula, and corpus callosum). None of the LITT cohorts demonstrated tumor location as a statistically significant predictor of increased adverse events or worse survival. However, the authors cautioned that lesions requiring a larger ablation zone, such as those near the eloquent cortex, could increase risk, meaning location may still have practical importance even if it was not statistically significant (35). PDT outcomes were influenced by proximity to SVZ with worse survival, stating it as a significant prognostic factor in at least one larger PDT cohort. Besides, PDT provides good local control but may be associated with higher rates of distant dissemination - a pattern that is especially relevant when tumors contact germinal zones (SVZ) or ventricles. Additionally, PDT’s limited light-penetration (~2–4 mm) constrains its use in deep or eloquent locations unless interstitial delivery is used, so location affects procedural feasibility and complication risk, for example, risk of edema/ICP rises after STR + PDT (32).

#### 4.3.4 Practical considerations

From a logistical standpoint, LITT requires intraoperative MRI or MR thermometry and a specialized team; it is minimally invasive and allows for relatively short hospital stays and repeat treatments when indicated (7, 40). PDT necessitates advance administration of a photosensitizer, detailed intraoperative planning for light delivery, and strict postoperative light avoidance to prevent systemic phototoxicity. Both techniques require interdisciplinary coordination and access to specialized technology (42).

When comparing PDT and LITT techniques, we observe that both present distinct therapeutic approaches. LITT, or magnetic resonance-guided LITT, works by inducing localized thermal necrosis in tumor tissue. On the other hand, PDT uses photosensitizers such as talaporfin sodium activated by laser irradiation, generating selective oxidative stress and inducing tumor cell death. Although the populations treated with both techniques show some homogeneity, typically patients with newly diagnosed or recurrent high-grade gliomas, tumor volumes vary depending on the studies and groups analyzed. For cases treated with LITT, median tumor volumes range approximately from 6 to 36 cm³, while patients undergoing resection followed by PDT present tumor volumes ranging from 6.1 to 30 cm³, with larger volumes generally associated with increased postoperative cerebral edema.

The adverse effects observed also differ to some extent between the two approaches. In LITT, the most frequent adverse events include cerebral edema, altered mental status, seizures, hemiparesis, intracranial hemorrhage, deep vein thrombosis, and bradycardia. In PDT, the notable adverse events include cerebral edema, altered mental status, cerebrospinal fluid fistula, infections, hemiparesis, cyst formation in the resection cavity, and episodes of delirium. Regarding prognostic parameters, OS shows variability, with medians ranging between 10 and 28 months depending on the study and population. PFS medians range from 2.8 to 16.4 months for LITT, and from 5.7 to 10.8 months for PDT.

A key difference between the techniques lies in local tumor control. LITT achieves significant tumor volume reduction through thermal ablation, whereas PDT is effective in eliminating residual tumor cells in the surgical bed after resection. However, studies indicate that although PDT is associated with lower local recurrence rates, it may show higher rates of distant tumor dissemination compared to control groups.

### 4.4 Limitations

The populations analyzed in the included studies were highly heterogeneous, encompassing pediatric and adult patients with high-grade gliomas of varying locations, tumor volumes, and functional status (KPS ranging from 70 to 100). This heterogeneity, combined with differences in adjuvant treatments, such as RT, TMZ, bevacizumab, or Gamma Knife, makes it difficult to isolate the specific effects of LITT or PDT on survival and disease progression. Moreover, studies assessed a wide variety of clinical outcomes, including PFS, OS, cerebral edema, intracranial pressure, local recurrence, and distant dissemination, limiting the ability to standardize measurements and perform robust quantitative synthesis.

Additional challenges arise from the biological characteristics of glioblastoma itself, including tumor heterogeneity and its immunosuppressive microenvironment, which may restrict the durability of immune responses induced by LITT or PDT. A minority of patients had IDH-mutant tumors, which do not meet WHO criteria for glioblastoma (2/16 in Quach et al., 2023; 1/10 in Peciu-Florianu et al., 2024) (44). Despite this, their inclusion is unlikely to substantially affect reported survival outcomes.

Finally, the lack of large-scale randomized trials comparing these therapies to standard treatments highlights the need for further research to validate their efficacy. Future studies should explore combinatorial strategies, such as LITT or PDT in conjunction with immune checkpoint inhibitors, to enhance antitumor immunity, particularly in light of PD-L1 upregulation observed post-LITT, which may suggest synergy with anti-PD-1/PD-L1 therapies.

## 5 Conclusion

Minimally invasive laser-based therapies, particularly LITT and PDT, offer promising adjuncts in glioma management, demonstrating favorable safety profiles and potential survival benefits. LITT is most effective in patients with small (<10 cm³), deep-seated, or unresectable tumors, especially when high ablation extents are achieved and KPS >70. Studies show LITT can achieve overall survival up to 16 months, comparable to repeat surgery, with the added benefits of shorter hospital stays and faster recovery. PDT, when combined with gross total resection, improves local control, reduces postoperative cerebral edema and intracranial pressure, and is associated with longer PFS and OS.

Despite encouraging results, current data are limited by small, heterogeneous cohorts and the absence of large randomized trials. PDT may carry a higher risk of distant dissemination, warranting careful patient selection and monitoring. The integration of LITT or PDT into multimodal regimens, including chemoradiotherapy, shows synergistic benefits and supports their role as effective adjuncts rather than stand-alone therapies. Future studies should focus on patient stratification by molecular profile and tumor characteristics to better define their optimal indications.

## Data Availability

The original contributions presented in the study are included in the article/**Supplementary Material**. Further inquiries can be directed to the corresponding author.
