# Supplementary material for: Emerging minimally invasive laser and light-based therapies for glioblastoma: a systematic review
Source: Front Oncol. 2026 Jan 20;15:1702399. doi: 10.3389/fonc.2025.1702399 (PMC12864125; doi:10.3389/fonc.2025.1702399)
Supplement: Supplementary file 1 [file Table1.docx]

***Supplementary Material***

# Supplementary Table 1. This table presents the PICOS strategy that specifies the Population, Intervention, Comparator, Outcomes, and Study design.

| **Population** | Patients diagnosed with glioblastoma |
| --- | --- |
| **Intervention** | LITT or PDT |
| **Comparator** | Surgery, biopsy, or no treatment |
| **Outcomes** | Overall survival (OS), progression-free survival (PFS), quality of life (QoL), tumor response, and adverse events or complications. |
| **Study Design** | Clinical trials or cohort studies |

# Supplementary Table 2. This table presents the tailored Boolean search strings used in each database (Embase, PubMed, Scopus, and Web of Science), along with the total number of results retrieved during the search period from January 1, 2020, to July 31, 2025.

| **Database** | **Search strings** | **Results** |
| --- | --- | --- |
| Embase | (( ( 'laser interstitial thermal therapy' OR 'interstitial laser thermotherapy' OR 'laser interstitial thermal ablation' OR 'laser interstitial thermo-therapy' OR 'laser interstitial thermoablation' OR 'laser interstitial thermotherapy' ) OR ( 'laser therapy' OR 'laser treatment' ) OR ( 'photodynamic therapy' OR 'photodynamic treatment' ) OR ( 'phototherapy' OR 'light therapy' OR 'photoradiant therapy' OR 'photoradiation therapy' ) ) AND ( ( 'glioblastoma' OR 'glioblastomas' OR 'gliobastoma multiforme' OR 'grade IV astrocytoma' ) )):ti,ab AND [2020-2025]/py | 375 |
| PubMed | TITLE-ABS ( ( ( {laser interstitial thermal therapy} OR {interstitial laser thermotherapy} OR {laser interstitial thermal ablation} OR {laser interstitial thermo-therapy} OR {laser interstitial thermoablation} OR {laser interstitial thermotherapy} ) OR ( {laser therapy} OR {laser treatment} ) OR ( {photodynamic therapy} OR {photodynamic treatment} ) OR ( {phototherapy} OR {light therapy} OR {photoradiant therapy} OR {photoradiation therapy} ) ) AND ( ( {glioblastoma} OR {glioblastomas} OR {gliobastoma multiforme} OR {grade IV astrocytoma} ) ) ) AND PUBYEAR > 2019 AND PUBYEAR < 2026 | 328 |
| Scopus | ((("laser interstitial thermal therapy"[Title/Abstract] OR "interstitial laser thermotherapy"[Title/Abstract] OR "laser interstitial thermal ablation"[Title/Abstract] OR "laser interstitial thermo-therapy"[Title/Abstract] OR "laser interstitial thermoablation"[Title/Abstract] OR "laser interstitial thermotherapy") OR ("laser therapy"[Title/Abstract] OR "laser treatment") OR ("photodynamic therapy"[Title/Abstract] OR "photodynamic treatment") OR ("phototherapy"[Title/Abstract] OR "light therapy"[Title/Abstract] OR "photoradiant therapy"[Title/Abstract] OR "photoradiation therapy")) AND (("glioblastoma"[Title/Abstract] OR "glioblastomas"[Title/Abstract] OR "gliobastoma multiforme"[Title/Abstract] OR "grade IV astrocytoma"[Title/Abstract]))) AND (("2020/01/01"[Date - Publication] : "3000"[Date - Publication])) | 333 |
| Web of Science | TS=((("laser interstitial thermal therapy" OR "interstitial laser thermotherapy" OR "laser interstitial thermal ablation" OR "laser interstitial thermo-therapy" OR "laser interstitial thermoablation" OR "laser interstitial thermotherapy") OR ("laser therapy" OR "laser treatment") OR ("photodynamic therapy" OR "photodynamic treatment") OR ("phototherapy" OR "light therapy" OR "photoradiant therapy" OR "photoradiation therapy")) AND (("glioblastoma" OR "glioblastomas" OR "gliobastoma multiforme" OR "grade IV astrocytoma"))) | 432 |
